# ‘Death on an industrial scale’- general practice trainees’ perceptions and experiences of dying and death during covid-19: an interpretative phenomenological analysis

**DOI:** 10.1186/s12909-024-06570-0

**Published:** 2024-12-23

**Authors:** Rebecca Holdsworth, Hugh Alberti, Bryan Burford, Emma Farrington, Gillian Vance

**Affiliations:** 1 Newcastle University, Newcastle upon Tyne, UK; 2 National Institute for Health and Care Research, London, UK

**Keywords:** Primary care, General practitioner training, Family medicine, Dying, Death, Palliative care, End-of-life care, COVID-19, Interpretative phenomenological analysis, Qualitative research

## Abstract

**Context:**

The COVID-19 pandemic led to an increase in numbers of patients dying at home in the UK, meaning that general practitioners (GPs) were exposed to more patient death than would be pre-COVID. This project aimed to gain insight into GP trainees’ experiences of patient death between March and July 2020. This insight can inform support for GPs, leading to improved wellbeing, workforce retention and ultimately, better patient-centred care.

**Methods:**

Interpretative Phenomenological Analysis (IPA) of semi-structured interviews was used to explore GP trainees’ experiences of patient death in one region of England.

**Results:**

Seven trainees, two male and five female, participated. They were working in both rural and urban community settings and were at different stages of GP training. Group experiential themes related to heightened emotional responses to patient death, managing uncertainty and the increased salience of relationships. Most appreciated positive teamworking and solidarity, though some had felt isolated within their surgery and wider community. There were some unforeseen positive experiences of individual and organisational healthcare changes, including a perceived new appreciation for the NHS workforce equality, diversity and inclusion (EDI) by actions to identify and reduce occupational hazards to at-risk healthcare staff. There were potential effects on career choice with participants feeling that changes during COVID-19 offered new flexibility in working arrangements and opportunity to sub-specialise within GP.

**Conclusion:**

More support to help navigate the amplified emotional responses to managing dying and death in the community is needed. Some experiences, particularly around managing uncertainty, can cause moral injury if not managed in a safe and supportive environment.

**Supplementary Information:**

The online version contains supplementary material available at 10.1186/s12909-024-06570-0.

## Introduction

The COVID-19 pandemic caused extreme physical and psychological pressure for medical staff across the world [1], whilst under the glare of media coverage and heightened public perceptions of the health service [2–4]. In particular, doctors found dealing with death in such high numbers to be challenging and overwhelming [5, 6]. More than two fifths of doctors in the UK felt that their levels of stress, anxiety and emotional distress had worsened since the COVID-19 pandemic began [7], with three quarters of General Practice (GP) trainees currently experiencing symptoms of burnout, stress, depression or anxiety [8–10]. At the same time, many GP trainees are planning on working less than full time after completing training [8, 11], while a survey of GP’s and GP trainees found that 42% said they were likely to leave the profession in the next five years because of the intensity of workload pressures [12, 13]. Over the last ten years, there have been some significant changes to how care of the dying person is managed in the community [14]. The GP workforce is already fragile, and a better understanding of how GP trainees navigate these difficult dying and death experiences is likely to inform educational strategies to improve this aspect of work, which may ultimately have benefit for the care of dying patients and support sustainability of the workforce.

### General practice training in the UK through COVID-19

General practitioner trainee/s (GPT) can be referred to as GP registrars (GPRs)/specialist trainees (ST)/doctors in training, primary care trainees/registrars/doctors in training and family medicine doctors. GP trainees are junior doctors who have completed medical school and foundation years (or equivalent) before starting three-years of GP specialist training (ST1/ST2/ST3). Two of the three specialist training years are completed in primary care (in England and Wales since 2021) [15].

End of Life Care (by which we mean patients likely within the last year of life) and/or care of the dying person (when a person is in the last days and hours of life) would typically include home visits or face to face consultations by the GPT, attending multi-disciplinary team meetings and co-ordinating management plans with hospital specialists and community nurses. During COVID-19, most GP and hospital consultations were held remotely by video or telephone, and referrals, investigations, meetings and follow-up appointments were temporarily suspended [16–18]. However, the approach to end-of-life care and care of the dying person was managed differently by GP surgeries, with some continuing face to face consultations and making home visits with adaptations, and others holding consultations via video calls or telephone [17].

### Patient dying and death through COVID-19

Deaths from all causes in the community during COVID-19 increased by a third compared to previous years [19]. Before COVID-19, most deaths in the UK (47%) occurred in hospitals, with around 23% occurring in private homes, 22% in care homes, 6% in hospices and 2% elsewhere. [20] In May 2020, during the ‘first wave’ of COVID-19 - equal numbers of patients died in hospital as care homes [21]. While dying at home (either in private homes or in care/nursing homes) is desired by many patients [22], through the COVID-19 pandemic, end-of-life care presented challenges for doctors [17].

During the peaks of the pandemic, GP trainees “stepped up” with “many running towards danger” [23] to deliver services, including caring for dying patients. Studies suggest that GP trainees felt adversely affected by changes to teaching and lack of day to day ‘non-pandemic’ GP exposure [6, 18]. Supervision from GP trainers continued but most formal teaching was disrupted and some GP trainees felt less supported, overwhelmed and lonely [6, 18]. There is some COVID-19 specific research on hospital staff and junior doctors general wellbeing and mental health [1, 2, 5, 7, 24], general experiences of healthcare workers [25], impact of doctors’ training [6], general primary care changes and innovations in end of life care [17, 18], and the educational impact on GPT [16], however, to the best of our knowledge, this is the first study focusing on doctors in training, specifically GPT, experiences of navigating dying and death during COVID-19.

Exploring GPT experiences of dying and death through COVID-19 has value beyond the pandemic context. At a time when UK healthcare and government leaders are aiming to improve care of the dying and encourage healthcare professionals (HCPs) to have earlier conversations about patients’ end of life decisions, it is important to ensure education and training aligns with the needs of HCPs working in the community [14, 26–28]. This study aims to addresses a gap in the literature by gaining insight into how GP trainees made sense of their experiences of caring for dying patients. This will help direct a focus on how educational systems can improve training and wellbeing support for GPT, with a view to benefiting workforce retention and ultimately patient care.

## Methods

The study adopted the methodology of Interpretive phenomenological analysis (IPA) [29]. This is an idiographic, hermeneutic phenomenological approach which examines how people make sense of major life experiences [30, 31]. Dying and death of a patient is a highly individual and personally significant experience, therefore ‘discovering’ the participants’ experiences requires an approach to make sense of their thoughts, feelings and attitudes to create their lived experiences [29, 32, 33]. As the subjects construct meaning from within collective unconscious experiences, this study aligns with a subjective epistemological position [31].

In IPA the researcher is engaged in a ‘double hermeneutic’, whereby the researcher interprets the participant’s own interpretation of their experience [29, 31, 32, 34, 35]. The lead author (RH) is a GPT who has experienced dying and death in the community during the pandemic. While some phenomenological approaches advocate ‘bracketing’, the separation of the researcher’s subjectivity and their interpretation of the data, IPA suggests that the researcher’s knowledge of, and engagement with the subject area can provide added weight and credibility to their interpretation [36].

### Ethics

A favourable ethical review was given by Newcastle University Faculty of Medical Sciences Ethics committee (2492/29356). Health Education England North East supported the study. Clinical trial number: not applicable.

### Recruitment

IPA recommends a small, relatively homogenous sample to provide insight into a particular individual experience [29, 31]. Purposive sampling by means of a screening survey distributed by the GP school via email to all GPT in the region identified those who were working in the community between March and July 2020 and had experienced patient death [31]. This timeframe was used as it was the start of the COVID-19 pandemic and captured the highest number of ‘excess’ deaths in April 2020 (43,796) in England and Wales [37]. The term ‘excess’ refers to the number of deaths above the five year average (2015–2019) [37]. Seven doctors were invited to take part in an interview to focus on exploring their experiences in-depth for a comprehensive understanding, instead of large numbers of participants which risks finding limited and superficial details.

### Data collection

Semi-structured interviews were conducted remotely via telephone (two interviews) or video (five interviews) due to the national restrictions in place at the time and participants preference of video or telephone [38]. Participants understood that participation was voluntary and informed, written consent was gained. They were encouraged to lead the conversation to explore areas more deeply that were important to them, with the interview schedule being designed to be flexible [39]. The interview schedule was loosely based around the research questions and current literature. It focused on their experiences of dying in the community setting, any other COVID-19 related dying experiences and comparisons to ‘pre- COVID-19’ dying experiences. The second part of the interview focused on how the GPT dealt with and learnt from their experiences and if there were any changes in perceptions.

RH conducted all interviews. Interviews lasted 45–60 min, and were digitally recorded, anonymised and verbatim transcribed by RH using TRINT [40] online transcription software. NVIVO 12 [41] was used to manage the interview data. Transcripts were sent to participants to check for accuracy. Participants’ wellbeing was checked periodically during the interview by having scheduled pauses and RH asking how they were feeling and whether they would like to continue or stop. They were sent a follow up email asking if there were any questions or comments as well as access to resources if they felt they needed support.

### Data analysis

All of the GPT narratives were analysed by RH, as outlined by Smith [29]. Personal experiential themes (PETs) for each participant were created by reading individual transcripts multiple times and constructing ‘clusters’ of interconnecting experiential statements. All authors then looked for similarities and differences, and points of convergence and divergence, across the contributing experiences, to create a set of Group Experiential Themes (GETs). RH, HA, BB, GV and EF contributed to the analysis, interpretation and editing of the narrative.

A record of all the research steps was kept. To support trustworthiness, a summary of themes was reviewed by a non-participant GPT/researcher as well as an academic medical peer. Reflexivity, being aware of one’s preconceptions that could lead inadvertently to influencing the outcomes, is integral to the research process [42]. RH engaged in reflexivity through collaboration with other researchers, who asked difficult questions about assumptions and decisions, and personal reflective writing of memos and journaling during the research process [43].

## Results

### Participants

Table 1 provides details of the participants’ backgrounds, which helped contextualise their responses, and informed interpretation.

#### Throughout the article, pseudonyms have been used to ensure anonymity

**Table 1:** GP trainees’ background

Participant pseudonyms	Participant backgrounds
Rosie	- GPST3 - UK graduate - White British female - Undertaking a palliative care integrated training post in secondary care then redeployed to a GP practice in a very socioeconomically deprived area.
Julia	- GPST3 - International medical graduate - Black African/British ethnicity female - Within her last month of GPT in a moderate socioeconomically deprived urban area
Ben	- GPST2* - UK graduate - White British male - Less socioeconomically deprived semi-urban area
Abeo	- GPST2* - International medical graduate - Black African male - Less socioeconomically deprived rural area
Olivia	GPST3, less than full time - UK graduate - White European female (Self described) - Moderate socioeconomically deprived urban area
Marie	- GPST3 - UK graduate - White British female - Pregnant and working from home as per UK government advice.
Kim	- GPST2* - UK graduate - White British female - Had started a six-month time out of programme experience, returned to GP training early to a less socioeconomically deprived rural area

*GPST2 at the time of interview, were GPST1 at the time of the first wave of COVID-19, which means they were reflecting on their early GP training experiences. The term GPST3 covers all GPT who are in their third year of training, however this may be longer than three years to accommodate for less than full time and integrated training posts, out of programme experiences or parental leave

### Group experiential themes

**Fig. 1:**
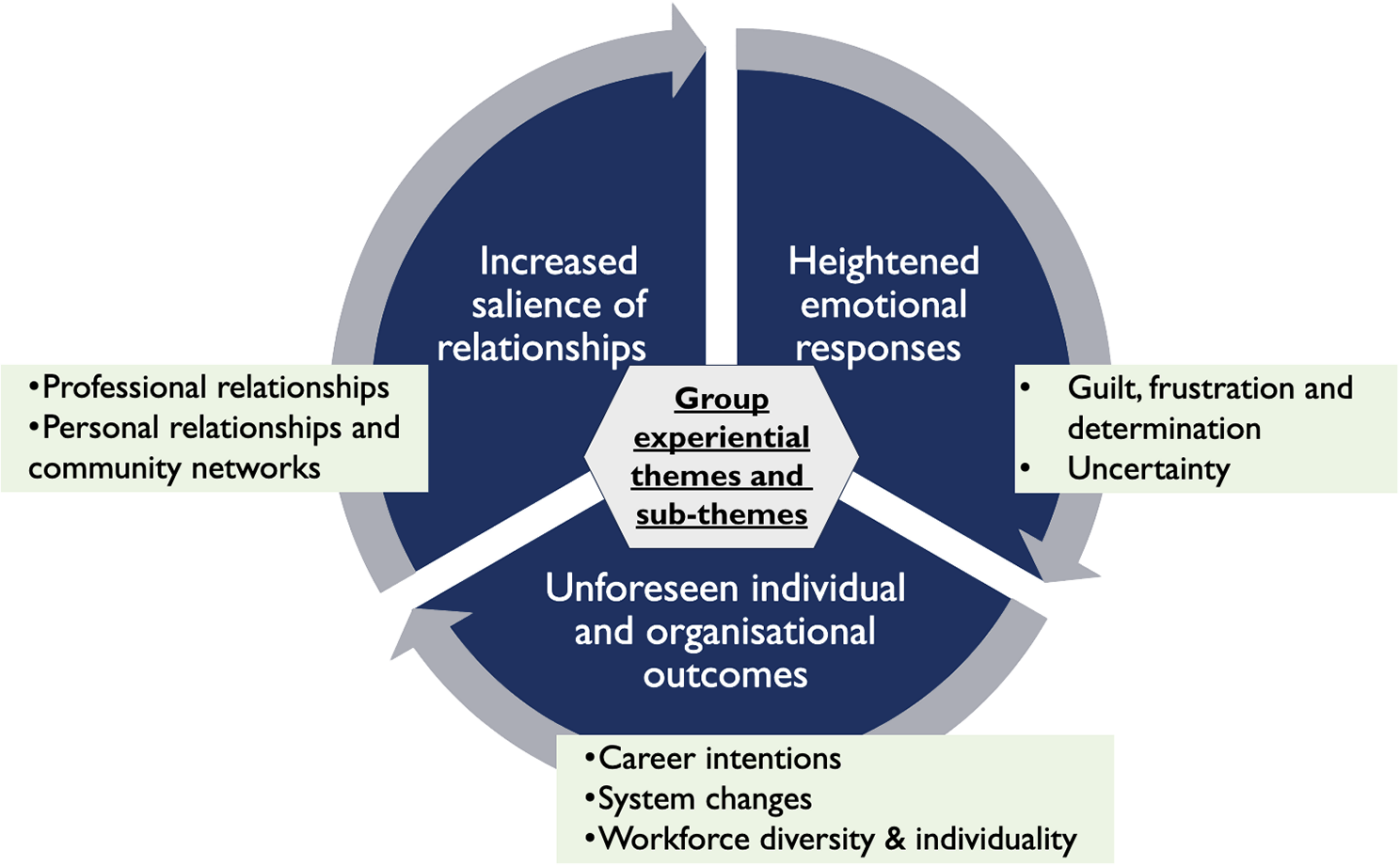
Group experiential themes and sub-themes

From the PETs, three Group Experiential Themes (GETs) with seven sub-themes were created (included in Fig. 1 below).

The extent of GPT experiences was illustrated by Ben who referred to death during COVID-19 being on an ‘industrial scale’. The cross-cutting themes discovered related to the heightened emotional responses, increased salience of relationships and unforeseen individual and organisational outcomes.

*‘During a pandemic, it’s well, it’s almost like death on an industrial scale… the fact that it’s [death] just so much more common and being done in a very different way, it’s almost like it feels a bit more matter of fact now, just, in general and I don’t think it should.’ Ben*.

### Heightened emotional responses

The rapidly changing context of COVID-19 medical management, the rising death toll and novel challenges led to a variety of amplified, intense emotional response compared to pre-pandemic dying and death experiences.

#### Guilt, frustration and determination

Olivia, Rosie, Abeo, and Ben felt that the restrictions of COVID-19 impacted on every part of end-of-life care and support, with fewer hospice beds, GP home visits and hospital specialist reviews. This led to feelings of guilt and frustration as it did not meet what they saw as being a standard of good care. These emotions can be defined as moral distress (psychological unease when professionals cannot take the morally correct action) and moral injury (sustained moral distress leading to guild and shame as well as betrayal and anger) [44]. However, Rosie was able to remind herself that despite the changed circumstances, the core of general practice was still there, meaning that she saw it was still possible to deliver good care to dying patients.

*‘I guess [its a] surprise that… in some ways… we can still execute bog standard general practice during a pandemic and people can have as close as possible… the experience they want of death and dying at home and it hasn’t completely rocked and destroyed that.’ Rosie*.

A bittersweet experience for Ben was dealing wholly remotely with a patient who later died as this had a powerful personal and professional impact on him. He felt guilty about not being able to care holistically for the patient and family as much as he normally would, whilst trying to detach to protect himself from the emotional dissonance. He felt valued by the family which was rewarding and made him determined to provide the best care for other dying patients going forward.

*‘It’s certainly one of those one of those things that… [will] stick with me forever… I diagnosed a chap was dying over the telephone and never actually saw him… it was upsetting personally, but also a little bit rewarding… because the family was so appreciative.’ Ben*.

#### Uncertainty

The challenges around how to care for dying patients were entwined with general uncertainties about medical care. Unfortunately, three of the participants had worked with staff-members who had died from COVID-19. Participants had the added dimension of transmission risk to consider, mostly for patients, but also for themselves. This caused some anxiety and influenced their decision making, for example whether it would be safe to complete a home visit. They felt an internal emotional conflict of how to support vulnerable patients safely, whilst protecting themselves.

*“What we were supposed to do in that circumstance? We didn’t feel that we really had any other option… I find it really difficult because I feel forced to be more distant from patients and from their family.”* Olivia.

Julia and Abeo discussed their ethnicity being linked to an increased morbidity and mortality risk.

‘It was scary… this GP [who died from COVID-19]… I could just see myself in her shoes… children, young, no medical conditions and… from BAME* background as well… it does drum home that message, that this is real, and you have to be careful… it was scary for… myself and [my] family.’ Julia.

“Forty-four…. doctors died and only one was white, the other[s were] within the BAME* group… it terrified me. I just know that I’m in danger.” Abeo.

* Julia and Abeo used the term ‘BAME’ (Black, Asian and minority ethnic), which was widely used at the time of the interview but is not the preferred term now.

One of Julia’s frail care home patients was transferred to the emergency department due to the carer’s uncertainty about how to care for dying patients with COVID-19. The patient was not in his familiar environment, despite this being his preference, in his final hours of life. This situation demonstrates the intricacies of pervasive uncertainty when managing dying and death in a COVID-19 pandemic context.

### Increased salience of relationships

Participants found different relationships offered support and comfort.

#### Professional relationships

Most participants had experienced positive teamworking and solidarity despite some isolating factors within GP surgeries, such as having to work alone or work from home, as well as the community-wide social distancing and lockdown measures. They found in-house and regional teaching and debriefing sessions to be a useful forum to share COVID-19 -related experiences and emotions of dying, as well as other unrelated subjects.

Three participants had felt isolated in their role and felt unable to discuss patients and those who were dying with their team easily. Olivia felt the loss of the usual connections with the practice staff due to a deliberate strategy to limit staff interactions to reduce spread of possible infection.

*‘I feel like I had very little kind of connection to the nursing staff or the admin[istration] staff….we’re encouraged not to have much contact with them…. [we can’t] just go and chat with them… it’s sometimes difficult to, to have that sense of teamwork.’ Olivia*.

Marie, who was shielding due to pregnancy and working from home, described a similar sense of isolation that resulted from the lack of direct in-person contact with colleagues. This was more challenging when having emotive conversations with dying patients and not being able to debrief or have a lighthearted chat with a GP team who understand.

Ben explained how he occasionally used alcohol to express his emotions when he was not able to reflect on some of his experiences because of cancelled teaching or supervision sessions.

*‘Obviously in an anonymised fashion… [I] have a chat about [the patient] with my partner [about] anything that is particularly bothering me or it ends up coming out when i’ve had a bit too much to drink.’ Ben*.

Olivia’s, Marie’s and Ben’s experiences show the importance of ‘normal’ professional contacts and emphasises the value of teaching sessions. When there was a lack of professional connection, GPT support needs were not always met and that lead to some GPT not coping well or struggling to process difficult experiences of dying.

### Personal relationships and community networks

Several participants talked about the importance of the emotional support they received from loved ones, who provided advice, and the physical and emotional time and space to offload their feelings.

However, the pandemic had a personal impact on Julia, Abeo and Ben, who lost friends and relatives during the COVID-19 pandemic. The parallel of caring for dying patients professionally and experiencing death personally meant they discussed feeling complex emotions including anger, frustration and sadness.

*‘Grieving for friends or family… it’s harder… than [grieving for] patient[s]… when friendship [or] blood is involved, it… drives it home… and [you] see yourself grieving over years.’ Abeo*.

On a wider scale, the GPT were grateful for practical support beyond the GP surgery, for example, the local fire service and schools provided practical help to supply PPE as well as messages of encouragement and motivation.

### Unforeseen individual and organisational outcomes

COVID-19 dying and death experiences made participants think more openly about their future careers and there were unforeseen individual and organisational changes within the healthcare system.

### Career intentions

The pandemic directly influenced three GPT career decisions mostly positively with some negatives. Rosie’s had considered leaving GP training to apply for palliative care specialty training prior to COVID-19 pandemic however, her experiences of caring for dying patients reignited her passion for GP-led palliative care.

*‘I kind of I started the job partly thinking about… leaving the GP programme to do palliative care training. And I think… [COVID-19 pandemic] made me want to be a GP who can do palliative care well… it’s better suited to what i want to do.’ Rosie*.

Olivia had been considering whether a career in General Practice could work for her family life or if an alternative career would be better, however she felt that COVID-19 introduced new opportunities in technology and innovation in GP surgeries. Changes such as remote consulting and more flexible working, compared to pre-pandemic helped her visualise working as a GP in different roles, for example, telephone consulting and out of hours work which includes urgent care for dying patients in the community.

*‘The job and the way that I’m working now bears no resemblance to… when I applied to become a GP all those years ago… that has definitely kind of raised lots of questions about what kind of work do…’* Olivia.

Kim had planned a six month ‘out of programme’ career break but as she was unable to travel during COVID-19 pandemic, Kim re-joined training as a second year GP registrar. Kim and her GP surgery experienced high numbers of COVID-19 deaths. It was a challenging time but she felt every experience of dying “teaches you… something new, something different”, which she wanted to learn from, to give patients and families a better experience of dying and death when she is a qualified GP.

### System changes

Some of the GPT reflected on the multiple clinical and legal updates, and changes in guidelines and processes as a result of the COVID-19 pandemic, such as changes in death certification. At times, the GPT found it overwhelming and impossible to keep up to date with the daily, weekly, monthly changes and regular inconsistencies. There were, however, some benefits as there was less administration which freed up time to focus on clinical and caring responsibilities, such as calling bereaved family members. Some changes streamlined processes that has been undisputed medical standards for decades, e.g. online coroner referrals, prescribing anticipatory medicines over the telephone. Some GP trainees questioned why a it took a COVID-19 pandemic to implement essential changes to modernise some of the day-to-day GP medical practices.

*‘The situation, the death certificate was a bit more straightforward because usually you already know this patient… the family, you know there are no concerns, and you go there and just see the body… it’s just so much time, energy and paperwork… [when] there’s no concerns… but nobody ever questioned it before COVID-19 happened.’ Julia*.

### Workforce diversity and individuality

An individual and organisational outcome from the COVID-19 pandemic for GPT has been the recognition that people from an ethnic minority have been disproportionally affected by the COVID-19 pandemic in many ways, including number of deaths. Julia felt valued as a staff member and felt nationally there was a positive move towards valuing diversity, especially in healthcare as more research and individual risk assessments were carried out with staff roles being amended if higher risks of morbidity and mortality were identified. The participants felt the NHS appeared to work together to value and support staff as individuals by reviewing those who possibly needed workplace adaptions e.g. ‘shielding’ pregnant staff like Marie.

Abeo and Julia had occupational health plans to reduce exposure and risk of COVID-19, however, at times the implementation of this was inconsistent, with community home visits being booked for them to high risk patients with respiratory symptoms when personal protective equipment was not available.

*‘When it is [a] grey [area]… it’s unclear and people push the boundaries… but I just took the precautions, and you try your best.’ Julia*.

## Discussion

This study explored how a group of GP trainees experienced dying and death during the COVID-19 pandemic. Analysis of these experiences identified three group experiential themes: ‘heightened emotional responses’, ‘increased salience of relationships’ and ‘unforeseen individual and organisational outcomes’. It is evident that these experiences had a profound impact on GPT wellbeing. However, there were many factors that helped them cope, and at times thrive, during this stressful and challenging time, as well as some concerns that could have a longer-term impact.

### GP wellbeing

Even before the COVID-19 pandemic, primary care doctors reported levels of distress linked to burnout, attrition and poorer quality of care [45]. During COVID-19, research suggests moral injury and suffering have been two concerning challenges reported by NHS healthcare workers and has been linked to post-traumatic stress disorder and depression [44, 46, 47]. Moral injury is a strong cognitive and emotional response following an “event which threatens one’s deeply held beliefs, trust, moral or ethical code” [46]. The BMA survey of UK doctors and existing research shows a correlation between moral distress and end of life care [44, 48]. The GP trainees’ range and depth of emotions demonstrated differing degrees of moral injury, distress, guilt and suffering, as well as internal coping mechanisms and external support from their relationships with others [46]. At times, the GP trainees’ emotions when caring for dying patients, encapsulated in the themes, demonstrated a level of stress and personal suffering from feelings of isolation, uncertainty and fear.

The participants approach to controlling strong emotional experiences is congruent with Gross’ conceptions of emotional regulation [47]. This concept broadly describes “five points in the emotion generative process, four of which are antecedent-focused, firstly, situation selection, secondly, situation modification, thirdly attentional deployment and fourthly, cognitive change, leading to ‘reappraisal’ before the emotional response, altering the entire subsequent emotional trajectory” [49]. “The fifth and final point is response-focused (experiential, behavioural or physiological) emotion regulation, a strategy of ‘suppression’ is a type of response modulation where an individual inhibits ongoing emotion-expressive behaviour” [49, 50]. Participants were mostly able, with time and support, to reduce the impact of uncontrollable factors of caring for dying patients during COVID-19, such as limited face to face interactions, communicating through PPE and visiting restrictions for relatives, and refocus on what they were able to realistically achieve like organising anticipatory medications and district nursing reviews, discussing the dying process and supporting relatives. Many of the system changes enabled positive work experiences that the GPT felt would benefit them day to day, akin to ‘reappraisal’ of Gross’s model by reducing the experiential and behavioural components of negative emotion. By reframing these processes, they were able to experience death and alleviate their distress through adapting to the ‘new normal’. This approach likely contributed to GPT having successful coping strategies, helping to build their resilience and manage situations with more control [47]. However, using individuals’ resilience as a concept can be criticised, as it can dismiss wider, structural issues and focus ‘blame’ on staff working in a dysfunctional system [51].

### GP training needs

Many aspects of caring for dying patients in the COVID-19 pandemic were different both personally and professionally but the experiences and learning are applicable beyond the constraints of the pandemic. Repeated experiences with multiple emotional conflicting ‘dissonance’ and contradictions, allowed for deeper understanding of how to approach and manage their dying and death encounters [52]. GP trainees become increasingly autonomous in their work over time as they grow in skills and confidence [53]. However, this growth in skills and confidence was accelerated compared to pre-COVID-19 pandemic training due to the increased volume of dying patients, which is evident from participants’ own reflections about dying and death. Although there was growth, there was grief, fear, and distress. None of the GPT appeared panicked through their reflections or from their professional actions despite the challenges. There was a lack of connection for the GPT at times because of the perceived and real distancing of the GP team and from dying patients. This impacted on the GPT ability to feel secure and gain the feedback and learning they desired. There were many points of progression towards more clinical responsibility during their experiences, which allowed for personal growth and professional development as well as causing guilt, frustration and uncertainty [16, 54]. For example, Ben, a second year trainee, felt very uncomfortable about diagnosing a patient as dying over the telephone, whereas Rosie, nearing the end of training, felt she was able to still provide good care for the dying despite the circumstances. A study with palliative care providers found that those who had been working in palliative care for longer had higher resilience, levels of self-care and self-compassion [55]. This might explain why the GPT participants who had just started training seemed to be more deeply and emotionally challenged by their experiences. Studies from previous pandemics and the COVID-19 pandemic have found many healthcare professionals experience ‘growth under pressure’ as well as developing more self-reflection and gratitude [24, 25, 56].

### Strengths and limitations

The study was conducted during, and with reference to, the extremes of the early pandemic period. While the exposure to death during that period may have been unusual, it is not completely removed from the experience of death during pre-COVID times. The volume of death may modify some experiences and emotional responses, but any of the participants’ accounts could equally have taken place before, or after the pandemic. Therefore, capturing and analysing these experiences are applicable and relevant to GP training at other times. As the GPT volunteered to be interviewed, they are self-selecting, motivated doctors, which could alter their experiences or imply more extreme views than their peers [57].

The participants found the opportunity to reflect and discuss what happened invaluable to process their experiences. It has highlighted the importance of discussing potentially emotive topics in research, if well-prepared and carried out carefully, as there can be unexpectedly positive outcomes for the individual participants.

### Implications for future practice and recommendations

This IPA research focuses on individual perceptions and experiences of dying and death; generalisation from these findings is not appropriate given the idiographic methodology. However, by considering the context of individual experiences, some transferability, to different people, different times and clinical contexts is possible [29]. Although not generalisable, this research demonstrates how these GPT adapted in highly unusual and unpredictable situations, especially when confronted with large numbers of dying experiences including personal losses. The findings affirm the value of GPT willingness to modify their approach to deliver holistic care for dying patients and their relatives, despite the challenges and adversity.

Going forward, GP trainees need to feel empowered with skills to ‘acknowledge, accept and regulate’ emotions [58]. Individual tutorials and teaching sessions with peers are valuable for sharing emotions and offer an opportunity to debrief in a safe space where GPT can receive support which, in turn, can reduce distress and improve wellbeing [47]. This aligns with other studies that suggest that support from colleagues, family and friends are an essential part of healthcare workers ability to cope [6, 17]. For UK-based GP trainees, regular small group teaching is valuable for professional and personal development [16, 59]. However, it is important to note that GPT who were shielding and working less than full time did not feel the same sense of support and felt increased isolation from their GP surgery and colleagues [18]. This has been shown in other studies and should be considered in planning flexible working arrangements for a diverse workforce [24]. Reducing administrative tasks and streamlining processes for dying patients and after death, supports good bereavement care as it frees time to follow up bereaved relatives and reflect and learn from the patients death [60, 61].

COVID-19 seemed to be a trigger for the NHS and wider communities to focus on confronting some equality, diversity, and inclusion (EDI) challenges [62]. EDI strategies need to be updated to reflect the experiences during COVID-19 and to find modern practical solutions to ensure fair treatment and training opportunities for all GP trainees.

## Conclusion

This study has shown the experiences and impact on GPT of dying and death in the community during COVID-19. More support for GPT to navigate the emotions of caring for dying patients is needed, particularly when managing uncertainty. Despite the challenging and novel circumstances of COVID-19, GPT were determined to be flexible and adapt to support dying patients and their relatives. The high volume of dying experiences allowed GPT to grow in autonomy and confidence if they were given a supportive space to reflect and debrief through peer teaching and individual tutorials. The COVID-19 pandemic is now officially finished but it continues to have a lasting effect generally on healthcare and its staff, as well as specifically for dying patients care in the community. The experiences during COVID-19 pandemic are highly relevant to the post-COVID-19 setting and will continue to inform General Practitioners and GPT care for dying patients. Further longitudinal, qualitative research with a larger group of GPT is needed to understand the long term personal and professional impact of dealing with dying and death to formulate appropriate educational strategies.

### Practice points

- While the volume of death during COVID-19 may modify some experiences and heighten emotional responses, these experiences are applicable and relevant to General Practice trainee dying experiences beyond the scope of COVID-19 pandemic.
- Support from colleagues, family and friends are an essential part of healthcare workers ability to cope.
- GPT have been committed to providing holistic care for the dying in uncertain and challenging situations and theorise that they gained strength from these experiences by reflecting, discussing, and adapting to thrive under the circumstances.
- Individual tutorials with GP supervisors and teaching sessions with peers are valuable to reduce suffering and improve wellbeing.

## Electronic supplementary material

Below is the link to the electronic supplementary material.

Supplementary Material 1

## Data Availability

No datasets were generated or analysed during the current study.
