# Supplementary material for: ‘Death on an industrial scale’- general practice trainees’ perceptions and experiences of dying and death during covid-19: an interpretative phenomenological analysis
Source: BMC Med Educ. 2024 Dec 23;24:1520. doi: 10.1186/s12909-024-06570-0 (PMC11668060; doi:10.1186/s12909-024-06570-0)
Supplement: Supplementary file 1 — Supplementary Material 1 [file 12909_2024_6570_MOESM1_ESM.docx]

BMC

Supplementary material: Interview Schedule

**Consent taken prior to the interview**

**Start of interview**

**Introduction:** Today we are going to discuss your experiences of dying and death during COVID-19 in the community as a GP trainee. It will involve talking about your experience as well around the general topics of COVID-19, dying and death and how it has affected you. There is no right or wrong answer, this is about ***your*** experience. Would it be ok to discuss these things?

**Background**

Age, Gender, Ethnicity, Stage of training, Location of GP practice

**Part 1: A. Dying and death experiences/Response to dying and death**

- If yes, please could I ask have you had any experiences of dying and death during COVID-19? (Likely answers are: yes, no, unsure)

- Do you mind sharing your experiences?

(Could structure as before, during or after)

- For the next questions I’m going to ask about a particular patient that comes to mind….

-What was the context/background? Where were you and where was the patient (e.g. home visit/telephone)?

-Had you met the patient before this? Was the patient expected to be dying/near death?

-How did you feel?

-What was your role?

-Who was involved? Professionals/MDT and family?

-Has this experience affected your relationship with your patient?

-What were the positives and challenges of this experience/situation?

-How do you think the dying/death was managed?

-Was this experience typical of your other dying and death experiences?

**B. Community setting**

-How do you think being in the community setting impacted on your experience of dying and death during COVID-19?

-Do you think it was different in other healthcare settings?

-Resources e.g. PPE, environment e.g. tent, deep cleaning.

-Different members of the teams, Teamwork, work load.- Stigma etc

**C. Non-patient/personal dying and death during COVID-19**

-Have you had any non-patient/personal experiences of dying and death during COVID-19  either of COVID-19  or non COVID-19?

-Were you or your family/friends/colleagues unwell with COVID-19? Did you have to self-isolate?

-(Ask any questions from previous section if relevant)

**Check on participant.**

**D. Dying and death experience during COVID-19 compared to previous experiences**

- How do these experiences compare to pre- COVID-19 experiences? Similarities or differences?

-professionally/ practically/ personally

**Check on participant.**

**Part 2: Learning/Impact of dying and death experiences**

Now I’m going to ask about how the experiences you’ve described have affected you professionally, and how you may have incorporated them into your professional learning.

**A. How do you feel that you have dealt with this/these experience/s?**

- Have your thoughts on the experience have changed over time?

- Have you reflected on these experiences? If so how (Talk it through with colleague/debrief domestically, written reflection, personal reflection or avoidance of thinking about experience?)

-Did any of the above help, or would you have liked another way to deal with your experiences? Can you explain what could have helped you deal with these experiences?

**B. What do you feel you have learnt from the experience?**

Were there learning points from this experience? Any surprise learning points?

What do you think impacted on your learning? Were there any barriers?

Did you have any teaching at the time/after the experience?

Do you feel that your emotional/personal response impacted on your learning?

**Check on participant.**

**C. Has this experience changed your perceptions about dying and death?**

Has this experience changed your understanding or interpretation of dying and death?

What do these experiences mean for you?

Do you think it will impact on your future practice? If so, how?

**To Finish**

Is there anything else about this/these experience/s of dying and death during COVID-19 that we have not talked about so far?

**What will happen next after the interview?**

I will send a follow up email with resources to support you if needed. Please remember to access support if this has been distressing or upsetting.

I will send a copy of the transcript to you to check over

I will send a copy of the final research project when it’s completed
